# Mast Cell Regulation of the Immune Response

**DOI:** 10.1097/WOX.0b013e3181c2a95e

**Published:** 2009-10-15

**Authors:** John J Ryan, Johanna K Morales, Yves T Falanga, Josephine FA Fernando, Matthew R Macey

**Affiliations:** 1 The Department of Biology, Virginia Commonwealth University, Richmond, VA

**Keywords:** mast cells, mast cell regulation, immune response

## Abstract

Mast cells are well known as principle effector cells of type I hypersensitivity responses. Beyond this role in allergic disease, these cells are now appreciated as playing an important role in many inflammatory conditions. This review summarizes the support for mast cell involvement in resisting bacterial infection, exacerbating autoimmunity and atherosclerosis, and promoting cancer progression. A commonality in these conditions is the ability of mast cells to elicit migration of many cell types, often through the production of inflammatory cytokines such as tumor necrosis factor. However, recent data also demonstrates that mast cells can suppress the immune response through interleukin-10 production. The data encourage those working in this field to expand their view of how mast cells contribute to immune homeostasis.

## Introduction

Mast cells are highly regarded for their important roles in atopic diseases such as asthma, allergic rhinitis, and atopic dermatitis. Their rapid activation by immunoglobulin E (IgE) crosslinkage and subsequent release of inflammatory mediators has been the subject of several recent review articles [1-3]. We are now appreciating that these cells have myriad functions in the immune response. In keeping with the long-standing theory that mast cells evolved as a means of protection from parasitic infection, these cells seem to be quite important as early sentinels of immune activation. However, this only scratches the surface of how mast cells can modulate inflammation. In fact, there is now compelling evidence that mast cells alters innate and adaptive immunity in ways that can either protect or damage the host. In this review, we will discuss the latest findings implicating mast cells in responses ranging from autoimmunity to cancer. Our goals are to reveal the broad impact mast cells have on immune homeostasis and to provide an update from the recent literature.

## Resistance to bacterial infection

In 1996, 2 groups made an observation placing mast cells squarely in the midst of bacterial immunity [4,5]. Put succinctly, mast cells are rapidly activated during bacterial infection and produce a number of mediators eliciting both innate and adaptive immunity. We now know that mast cells express an array of innate immune receptors including members of the Toll-like receptor (TLR) family and complement receptors as reviewed by Sayed et al [6]. The role of mast cell activation in this process is currently being clarified and appears to be nuanced in important ways that have therapeutic implications.

The greatest risk of bacterial infection remains sepsis, with resulting shock and loss of organ perfusion that is life threatening. Mouse models of bacterial infection have demonstrated that an absence of mast cells greatly increases the death rate because of septic peritonitis. The initial reasoning for this was that mast cell activation by bacteria led to the secretion of tumor necrosis factor (TNF), recruiting neutrophils to the site of infection [4,5]. Although this certainly occurs, there are several other factors involved that have only recently been elucidated. The current thinking is that mast cell activation leads to the rapid release of TNF and other factors that blunt infection. In addition to eliciting neutrophil migration to the site of infection, mast cell-derived TNF evokes dendritic cell trafficking to draining lymph nodes. The subsequent activation of T-cells by dendritic cells results in the necessary hyperplasia prompting a full and productive adaptive immune response. In the absence of mast cells or TNF, lymph node hyperplasia is greatly diminished and mice are much more susceptible to bacterial infection [7]. However, mast cell-derived interleukin (IL)-6 and the mast cell protease (mMCP)-6 are also clearly important because loss of these proteins also increases susceptibility to infection [8,9]. Like TNF, it appears that these enzymes are also involved in the necessary recruitment of neutrophils to the site of infection. Recent work has further demonstrated that MCPs can protect the host from hypotensive shock by degrading the peptides endothelin-1 and neurotensin [10-12]. Therefore, it appears that mast cells have a direct impact on immune responses to bacteria and also modulate changes in the vasculature, preventing pathologic responses to pathogens.

It is important to note that bacterial infection studies have been carried out in mouse model systems. However, the data are consistent in differing assay systems and in both the gastrointestinal and pulmonary systems. The pathogens tested include *Escherichia coli, Staphylococcus aureus, Mycoplasma pulmonis, Haemophilus influenzae, Klebsiella pneumoniae, Citrobacter rodentium, Helicobacter felis*, and *Psudomonas aeruginosa *[13]. As such, the role of mast cells in protection from bacterial infection appears quite important.

### Mast Cells and Autoimmunity

In addition to their well-documented role in immediate hypersensitivity (type I hypersensitivity responses), recent studies have found mast cells functioning in the development of autoimmune diseases classically termed types II-IV. A consistent theme in these studies is the ability of mast cells to elicit chemotaxis of other immune effector cells that sustain the disease, reminiscent of the role mast cells play in asthma. We will discuss each of these subtypes separately.

### Type II Autoimmune Diseases

In bullous pemphigoid (BP) and Graves' disease (GD), the type I predilection of mast cells may contribute to the progression of these type II autoimmune diseases. Mast cells and IgE have been implicated in autoimmune pathology, able to reproduce the early phase of BP lesion development in human skin grafted to nu/nu mice [14,15]. Indeed, human BP is associated with elevated serum levels of IgE autoantibodies and the presence of eosinophils in blisters, supporting a role for IgE acting through mast cells in human BP [16,17].

In the murine BP experimental model, mast cells have been shown to play a key role in neutrophil recruitment. That is, mast cell degranulation occurs within ~60 minutes of antibody transfer, causing neutrophilic infiltration and subsequent blistering of the skin [18]. Mast cell-deficient mice or wild-type mice treated with an inhibitor of mast cell degranulation both fail to develop the disease [19]. Local engraftment of W/W^v ^mice with wild-type bone marrow mast cells (BM-MCs) restores the BP phenotype, confirming the role for mast cells [18]. These authors postulate that mast cells triggered by complement activation may be a crucial source of CXCL8, a potent neutrophil chemoattractant.

Mast cells also play a role in GD ophthalmology, where they infiltrate the orbital tissue of the eye and precede the appearance of lymphocytes [20]. Further, increased circulating levels of stem cell factor (SCF) and IgE antibodies, some of which are thyroid stimulating hormone receptor-specific, are observed in a GD cohort [21]. Mast cells are theorized to function in GD ophthalmology as important sources of chemoattractants (IL-16, CCL2), cytokines (IL-4, IL-5, IL-13), and B cell costimulatory signals (CD154) [22].

### Type III Autoimmune Diseases

Mast cells have been documented to function in type III autoimmune diseases, including the Arthus reaction, systemic lupus erythematosus (SLE), and rheumatoid arthritis (RA) as reviewed by Sayed et al [6]. Mast cells were first implicated in a cutaneous Arthus response using W/W^v ^mice in 1991. When compared with wild-type controls, mast cell-deficient mice showed decreased edema, neutrophil infiltration, and hemorrhage [23]. Activation of the mast cell through Fc*ε*RIII and complement, resulting in TNF production and neutrophil recruitment, was found to be important for this response [24-26].

The role of mast cells in SLE is less clear. Previously, Hiromura et al showed that mast cell infiltrates are present in affected tissue from patients with SLE-mediated glomerulonephritis, suggesting a mast cell influence [27]. This theory was supported by findings from Lyn kinase-deficient mice, which develop lupus-like inflammation and demonstrate mast cell hyperresponsiveness [28,29]. However, a pristane-induced model of SLE showed that mast cell-deficient W/W^v ^mice still develop SLE symptoms that are equal or greater in magnitude compared with wild-type littermates [30]. This suggests that mast cells have no role in SLE disease or may actually exert a protective influence. It is important to note that SLE can develop through several mechanisms that converge on a similar destruction of joints, kidneys, and other organs. It may be that the importance of mast cells varies with lupus etiology.

In RA patients, the arthritic synovial tissue shows increased mast cell numbers and includes mast cells that seem to be degranulated [31]. High levels of tryptase and histamine have also been detected in the synovial fluid of some RA patients and implicated in murine models [31,32]. Mast cell-deficient mice do not develop disease in an autoantibody model of RA, whereas disease was restored when the mast cell compartment was reconstituted [33]. This seemed logical, because mast cell-derived TNF has been implicated in disease pathogenesis, and anti-TNF therapies are efficacious in human RA [34]. The ability of some TNF-null mice to develop arthritis after serum transfer suggests that other mediators are required for RA disease progression, and that these mediators may act in association with TNF. In support of this, mast cells secrete IL-1 in the serum transfer model via Fc*ε*RIII-mediated activation, and IL-1 completely restores arthritic disease in W/W^v ^mice [35,36]. These findings offer new hope for treatments targeting not only inflammatory cytokines, but mast cells. For example, imatinib mesylate, which inhibits Kitmediated mast cell survival, effectively prevents collagen-induced arthritis development and also treats established disease [37]. It is important to note that imatinib mesylate suppresses BCR-Abl and other tyrosine kinases, and is therefore neither mast cell-specific nor free of side effects. However, this drug and others like it offer hope for progress in suppressing mast cell responses.

Despite the logical nature of these findings, the importance of mast cells in RA has been recently debated. Work from the laboratory of Howard Katz showed that mast celldeficient W^sh^/W^sh ^mice develop arthritis similar to wild-type littermates after anticollagen antibody injection [38]. It appears that W/W^v ^mice have a neutrophil deficiency that has been underappreciated and that neutrophils rather than mast cells may be the key determinant in at least this model of RA. As with all animal models, translation to human patients remains an important hurdle, but our fundamental understanding of disease onset and progression is constantly evolving in ways that will certainly provide patient benefit.

### Type IV Autoimmune Diseases

Much work has been devoted to elucidating the mast cell's role in type IV autoimmune diseases, with the best-described data related to multiple sclerosis (MS). Mast cells have been shown to accumulate at sites of inflammatory demyelination in the brain and spinal cord and are often found there in a degranulated state [39]. Furthermore, high levels of tryptase and histamine are often found in the cerebrospinal fluid of MS patients, suggesting mast cell activation [40,41]. Gene-expression profiling has demonstrated that transcripts encoding the histamine 1 (H1) receptor, tryptase, and Fc*ε*RI, are highly expressed in the central nervous system (CNS) plaques of chronic MS patients [42].

W/W^v ^mice display a very mild MS disease state and show delayed onset when compared with their wild-type littermates [43]. Furthermore, the entry of CD4^+ ^and CD8^+ ^T-cells into the CNS also appears to be compromised in W/W^v ^mice [44]. Indeed, several aspects of the T-cell response to myelin peptide in MOG_35-55_-induced experimental allergic encephalomyelitis (EAE) seem to be defective in W/W^v ^mice. Selective mast cell reconstitution of W/W^v ^mice through IV transfer of BMMC restores severe disease susceptibility, [44] strongly supporting a role for mast cells.

How mast cells promote EAE is a developing story. It has been suggested that MCPs expressed in the CNS could contribute to direct local tissue destruction. Their presence in areas prone to autoimmune damage, including joints, the CNS, and the pancreas, is consistent with this idea [45]. However, mast cells may not exert their most potent effects on MS from within the CNS. Tanzola et al suggest that mast cells act outside the CNS, because mast cell reconstitution does not appreciably repopulate the brain or spinal cord [46]. Thus, the current thinking is that mast cells promote MS by inducing migration of inflammatory cells into the CNS. Gregory et al showed that W/W^v ^mice exhibit 5- to 7-fold decreases in the number of CD4^+ ^and CD8^+ ^T-cells that enter the CNS, corresponding with reduced inflammation and demyelination in the brain and spinal cord. Furthermore, CD8^+ ^T-cells primed in mast cell-deficient mice express significantly decreased amounts of interferon (IFN) and CD44 post-MOG immunization, despite normal T-cell development and comparable peripheral T-cell numbers in these W/W^v ^mice [44]. These data fit the known role for mast cells in cell recruitment. That is, mast cells are known to elicit migration of many cell types, including myeloid dendritic cells and effector CD8^+ ^T-cells, through the release of factors including LTB_4 _and TNF [47,48].

These results suggest that mast cell-mediated effects on T-cells may be paramount in type IV hypersensitivity. Oddly, mast cell-derived IL-4, a T_H_2 cytokine, appears to be one of the major players in the progression of MS, a T_H_1- and Th17-associated disease. Mast cell-derived IL-4 is necessary for severe disease progression and to produce an optimal T_H_1 response in MOG_35-55_-induced EAE [49]. Yao et al explain this paradox by showing that IL-4 promotes IL-12 expression through the inhibition of IL-10 transcription in dendritic cells [50].

How mast cells are activated in MS is being clarified. Melissa Brown's group has demonstrated that Fc*γ*R-mediated activation can be a critical component to EAE [51]. However, it is also possible that complement receptors play a vital role. Urich et al recently showed that EAE exacerbation by injecting antimyelin oligodendrocyte protein antibodies was dependent upon complement [52]. Because mast cells express several complement receptors, including C3aR, C5aR, CR2, and CR4, [53] activation by more than one pathway seems quite plausible.

Collectively, these studies strongly suggest a role for mast cells in MS elicitation. Many MS patients suffer from a relapsing and remitting form of the disease. One wonders if the numerous therapies used to combat the prototypical mast cell-associated diseases, allergy, and asthma, might be efficacious at least in lengthening remission. These could include histamine- and leukotriene-targeted agents. LTB_4 _antagonism has been used successfully in EAE models [54,55]. The role of histamine in MS is difficult to discern from the literature. For example, the use of H1 antagonists appears to delay the onset of multiple sclerosis symptoms, [56] and suppressed EAE severity [57]. Likewise, the H2 antagonist dimaprit inhibited EAE pathology, including suppressing blood-brain barrier leakage [58]. These data suggest that histamine may promote MS pathology, perhaps because of its vascular effects. However, histamine decarboxylase-deficient mice, which cannot make histamine, have more severe EAE, indicating a protective role for histamine. Related to this, loss of histamine H3 receptor expression, which is normally confined to the nervous system, also appears to exacerbate EAE [59]. A unified view is that histamine interactions with its H1 and H2 receptors promotes inflammation and vascular leakage, whereas H3 interactions in the CNS are protective.

### Mast cells and inflammatory bowel disease

The role of mast cells in inflammatory bowel disease (IBD) has been the focus of a recent review [60] and will be dealt with briefly here. IBD is often categorized into 2 major subcategories: Crohn's disease, which is largely classified as a T_H_1 inflammatory condition, and ulcerative colitis, typically classified as a T_H_2 condition. The evidence that mast cells participate in IBD is logical but largely correlative. Mast cells are found in the gut, particularly in the lamina propria and the submucosa, located near blood vessels and nerve endings [61-66]. Their activation is known to elicit mucosal exudation, leukocyte recruitment, and interactions with the nervous system [26,67-70]. Therefore, mast cell involvement in IBD has been strongly suspected, especially given that mast cell numbers and mediators are increased in the gastrointestinal tract of IBD patients [63,71-74]. Further data suggest that mast cells are an important source of TNF in IBD, and that steroids or TNF blocking antibodies can reduce IBD severity in part by blocking mast cell-derived TNF [75-81].

As described with clarity by Rijnierse and coworkers (2007), there are discrepancies concerning the role of mast cells in IBD when mouse models are employed. Two issues need to be taken into account. First, some animal models employ inflammatory substances such as 2,4,6-trinitrobenzene sulfonic acid or Dextran sodium sulfate that are either dissolved in ethanol-containing solutions or directly elicit epithelial damage. These models have demonstrated disease without evidence for a mast cell component. Secondly, some experimental models have employed Ws/Ws rats that are purportedly mast cell deficient. As discussed by Rijnierse and coworkers, there is evidence that these animals in fact do have mast cells in the colon, making it difficult to interpret data using Ws/Ws rats. These authors have recently developed a hapten-based model of colitis that does not occur in mast cell-deficient W/W^v ^mice, and demonstrates a role for mast cell-derived TNF in disease pathology [82]. We look forward to learning more about the role of mast cells in this chronic inflammatory disease as relevant models progress.

### An unexpected suppressive role for mast cells in type IV hypersensitivity

Although mast cells are most often noted for their inflammatory capacity, recent data suggest that they are not monolithic. For example, Hagaman and coworkers showed that human mast cells can secrete IL-1 receptor antagonist, a plausible means of suppressing inflammation [83]. Striking evidence of mast cell-mediated immunosuppression comes from recent work published by the laboratory of Stephen Galli [84]. In studying type IV hypersensitivity reactions to poison oak and poison ivy, this group found that mast cell-deficient mice (both W/W^v ^and W^sh^/W^sh^) exhibited stronger responses than wild-type littermates to uroshiol, the allergen-bearing sap derived from poison oak and poison ivy. A normal inflammatory response was found when wild-type mast cells were transplanted into mast cell-deficient mice. Conversely, mice reconstituted with IL-10^-/- ^mast cells showed increased epidermal ulceration and necrosis and dermal leukocytic infiltration compared with controls, showing that mast cell-derived IL-10 is required for limiting inflammation and edema associated with urushiol. Lastly, engraftment with FcR*γ*^-/- ^BMMC, which lack expression of both Fc*ε*RI and Fc*γ*RIII, failed to restore the suppressive effect, indicating that mast cells are activated through Fc receptors in this model [84]. The ability of mast cells to limit inflammation, through IL-10 secretion or other factors, creates a nuanced picture of the mast cell response warranting reconsideration in some cases.

### Mast Cells and Cancer

The link between inflammation and cancer is a long-standing observation now supported by substantial scientific evidence. In fact, many cancers are associated with specific inflammatory conditions. For example, colorectal cancer is associated with IBD, Crohn's disease, and chronic ulcerative colitis. Pancreatic carcinoma is linked to chronic pancreatitis, whereas lung carcinoma is associated with bronchitis [85]. Additionally, infectious agents causing chronic inflammation are known to increase cancer incidence. *Heliobacter pylori *is the world's leading cause of gastric cancer. Similarly, Hepatitis B and C viruses increase the incidence of hepatocellular carcinoma [85,86]. The fact that long term use of nonsteroidal anti-inflammatory drugs, including cyclooxygenase (COX) inhibitors, greatly reduces the risk of colon cancer and breast cancer illustrates the strong link between inflammation and cancer is [85,87-90].

If chronic inflammation promotes oncogenesis, the mast cell is a logical participant in this process. Mast cells potentiate inflammation by releasing mediators such as histamine, leukotrienes, tryptase, and prostaglandins, which collectively increase vascular permeability and promote leukocyte migration. Mast cells also produce angiogenic and inflammatory factors, including vascular endothelial growth factor (VEGF), monocyte chemotactic protein-1 (MCP-1; CCL2), MCP-2 (CCL8), monocyte inflammatory protein-1*α *(MIP-1*α*; CCL3), IL-4, IL-13, IL-1*β*, granulocyte macrophage colony stimulating factor (GM-CSF), granulocyte colony stimulating factor (G-CSF), IFN-*γ*, and TNF [2,85,91]. Please note that the set of cytokines produced varies with the mast cell-activating stimulus, and the species studied. Importantly, significantly increased numbers of mast cells have been found at the sites of many human and murine tumors, including malignancies of the breast, pancreas, lung, and stomach [92-96]. Mast cell migration is most likely mediated by SCF, which is secreted by many tumors and promotes mast cell activation [2,97-99]. Increased mast cell numbers have also been observed in patients with chronic inflammatory conditions known to promote cancer, including *H. pylori *infection [100] and IBD [72,101]. Mouse experiments have shown increased mast cell numbers associated with skin carcinogenesis [102,103] and chemically induced intestinal epithelial tumors. Furthermore, tumor incidence was significantly decreased in mast cell-deficient c-*kit *mutant mice [104]. Similarly, mast cells are necessary for the initiation of adenomatous polyps in the colons of mice predisposed for this condition [105] and for the expansion of pancreatic islet tumors resulting from aberrant expression of the Myc transcription factor [106].

**Figure 1:**
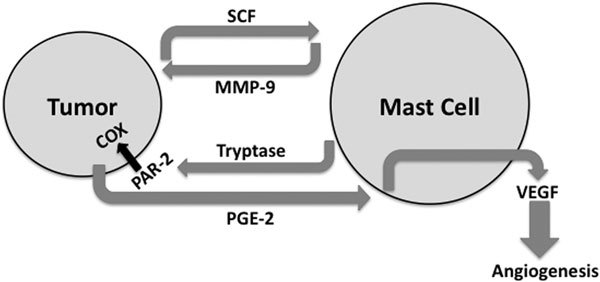
**Positive feedback loops promoting mast cell-mediated angiogenesis in cancer**. A wide variety of tumors are known to secrete SCF, which induces mast cell migration in vivo. Mast cell activation by SCF induces MMP-9 production, which clips membrane-bound SCF from the tumor cell surface, increasing the bioavailability of SCF. Activated mast cells produce tryptase, which can enhance tumor cell COX activity via the PAR-2 receptor. COX-mediated PGE-2 production can activate mast cells to secrete VEGF, promoting tumor angiogenesis.

It is plausible that mast cells promote tumor angiogenesis by secreting VEGF as part of an inflammatory cascade (Figure 1). Tryptase, secreted by activated mast cells, activates the PAR-2 receptor, increasing COX activity [107-109]. COX activity prompts PGE2 production, which is known to elicit VEGF production in mast cells [90,110]. Human mast cells are capable of secreting all 4 isoforms of VEGF-A, and VEGF-B, VEGF-C, and VEGF-D [110,111]. Mast cells have also been shown to be pivotal in the angiogenesis of several murine tumors [102,106]. VEGF, also secreted by tumor cells, is known to have a highly mitogenic effect on endothelial cells, thus contributing to the neovascularization critical for tumor growth and survival [111-113]. It is noteworthy that mast cells also express VEGFR1 and VEGFR2, making it possible for tumors and mast cells to use VEGF in both autocrine and paracrine manners [110]. Another mediator of inflammation is TNF, which effectively recruits neutrophils and macrophages. TNF has been shown to increase angiogenesis, tumor growth, and metastasis [114]. Like VEGF, TNF can also be secreted by both mast cells and tumor cells [1,29,30]. Therefore, it is plausible that crosstalk between mast cells and tumor cells creates a positive feedback loop exacerbating inflammation, promoting malignant transformation and tumor progression.

A recent paper by Huang et al [115] emphasized the potential of mast cell involvement in tumor formation. This group demonstrated that a wide array of tumor types secrete SCF, which promotes mast cell recruitment to tumors in vivo. Furthermore, the presence of mast cells in the tumor enhanced tumor growth and decreased host survival. Although SCF is known to promote many activities in mast cells, an interesting issue noted in this paper was SCF-induced secretion of matrix metalloproteinase (MMP)-9 secretion. MMP-9 furthers SCF production from tumors by cleaving surface-bound SCF into a soluble molecule, facilitating a positive feedback loop (Figure 1). An important aspect of this work was the indication that mast cells may promote immunosuppression at the tumor site, perhaps because of the increased presence of regulatory T-cells. We found these data to be particularly intriguing, because of our recent demonstration that mast cells can cause regulatory T-cell migration [116]. Collectively, this group and the work of others suggests that mast cells may be a critical player in tumor progression, promoting angiogenesis and immunosuppression. Certainly, more work in this important area is warranted.

### Mast Cells and Sclerosis

#### Systemic sclerosis

Sclerosis, the hardening of tissues, affects several different organs. There is evidence that mast cells can contribute to sclerotic pathology. The role of mast cells in systemic sclerosis (SSc) has been covered in 2 recent review articles [6,117] and will be dealt with only briefly here. SSc is denoted by autoimmunity, inflammation, and vascular pathology with progressive interstitial and perivascular fibrosis that can affect the skin and many internal organs. The disease is considered incurable and in its most severe form (diffuse cutaneous SSc) has a mortality rate of 45% in 10 years [118,119]. Mast cells are known to secrete TGF*β *and proteases that collectively promote sclerosis [120]. A mouse model employing so-called tight skin mice has demonstrated an increase in mast cell density and degranulation [121]. This correlation is strengthened by data indicating that mast cell stabilizers or chymase inhibition can improve disease in these animals [122-124].

#### Atherosclerosis

The development of atherosclerosis is caused by the activation and dysfunction of endothelial cells. A local defect in endothelial cell homeostasis promotes the adhesion of leukocytes and activated platelets to the damaged endothelium. This leads to increased vessel permeability that is promoted by lipid components in the plasma, such as oxidized low-density lipoprotein (oxLDL). Macrophages exposed to oxLDL differentiate into foam cells [125]. Foam cells secrete a variety of substances involved in the early phases of plaque development in the vessel intima, known as fatty streaks. The development of fatty streaks is facilitated by diverse leukocytes, including neutrophils, monocytes, macrophages, basophils, B- and T-cells, dendritic cells, and mast cells [125]. These inflammatory cells promote an accumulation of lipids via increased blood vessel permeability, enhancing the maturation of fatty streaks into mature atherosclerotic plaques. Plaques are covered by an inflammatory cap containing macrophages, T-cells, and mast cells. Over time, the growing plaque narrows the blood vessel lumen, resulting in locally increased blood pressure upstream from this stenosis. Eventually, the plaque can rupture. Release of tissue components into the blood initiates the coagulation cascade and the subsequent formation of a thrombus. The thrombus travels through the blood vessel and can cause arterial occlusion, resulting in complications such as pulmonary emboli, myocardial infarction, or stroke [125,126].

Several studies have demonstrated mast cell accumulation in both human and mouse atherosclerotic plaques, perhaps 6-fold greater than normal tissue [125-128]. How mast cells are activated in this condition remains unclear; a simple explanation may be the increase in hydrostatic pressure at the site of the plaque. Activated mast cells in the plaque can contribute to atherosclerosis in several important ways. Their cytokine and chemokine secretion, particularly TNF and IL-6, promotes leukocyte infiltration. Furthermore, histamine and lipid-derived factors can act as autacoids, regulating vascular tone. Mast cells also produce proteases, which modify vascular permeability and remodeling. Particularly, serine proteases derived from mast cells, such as chymase and tryptase, can activate MMPs locally, causing plaque instability because of decreased thickness [129]. Mast cells are likely contributors to plaque neovascularization, which initially promotes tissue survival. However, these vessels are fragile, and their rupture can facilitate plaque release [130].

Recent advances in this area include work from the laboratory of Guo-Ping Shi [131]. This group used an atherosclerosis-prone LDL receptor-deficient mouse to demonstrate that mast cell deficiency (via crossing to W^sh ^mice) reduces lesion size, clinical score, lipid content, and the number of T-cells and macrophages present. They also noted an increase in collagen content and cap size, which could stabilize the plaque. Using mast cell reconstitution, this group further demonstrated that IL-6- or IFN*γ*-deficient mast cells did not restore atherogenesis like wild-type mast cells, suggesting that mast cell-derived IL-6 or IFN*γ *enhances plaque formation. An interesting aspect of these studies was that TNF-deficient mast cells did restore atherogenesis, suggesting that mast cell-derived TNF was not essential to this process. The authors further showed that proinflammatory cytokines such as IL-6 are likely acting to increase the expression of cysteine protease cathepsins, which degrade the connective tissue matrix and promote atherogenesis.

## Conclusions

**Figure 2:**
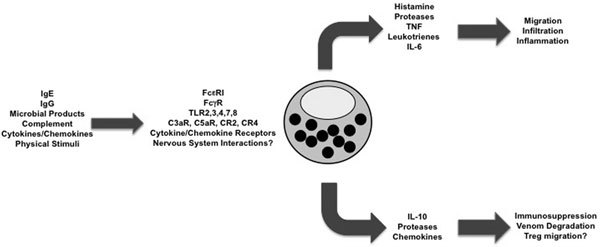
**A summary of mast cell pro- and anti-inflammatory activities**. Mast cells are activated by a wide array of stimuli, consistent with a sentinel role. However, mast cell activation can both enhance and inhibit inflammation, a process that is not fully understood and likely involves significant contextual cues. Of the many possible factors, TNF appears to be the most common in eliciting inflammation, whereas IL-10 is a logical and powerful immunosuppressant.

Mast cells are now appreciated as a type of frontline sentinel, activated by numerous stimuli and employed to protect the host in a variety of situations (Figure 2). This role in innate immunity is augmented by their ability to interact with and alter the adaptive immune response. Unfortunately, the mast cell response can be pathologic as well. Although a number of mast cell activities warrant focus, a recurring theme is their ability to secrete chemoattractants such as TNF, IL-6, leukotrienes, and some proteases. These factors, working together with the vasodilatory effects of histamine, prompt cellular movements toward target organs, even to tissues where the activated mast cell is not located. An interesting and new aspect of mast cell biology is their apparent ability to provide immunosuppression. Although IL-10 production is a logical mechanism for this function, the role of proteases in degrading venoms and regulating shock, and the unidentified factor(s) promoting regulatory T-cell migration, seem to be important. A wonderful aspect of the long history of mast cell-associated atopic disease is our possession of efficacious clinical tools targeting mast cell function. We look forward to learning how these tools can be employed in nonatopic conditions where mast cells have a role.

## End Note

Supported by National Institutes of Health Grants 1R01-AI-059638 and U19-AI-077435 to J.J.R
