# Mast Cell Regulation of The Immune Response: Erratum

**DOI:** 10.1097/WOX.0b013e3181d1d60f

**Published:** 2010-01-15

**Authors:** John J Ryan, Johanna K Morales, Yves T Falanga, Josephine FA Fernando, Matthew R Macey

## 

In the article "Mast Cell Regulation of the Immune Response", which appeared in volume 2 of *The World Allergy Organization Journal *on pages 224-232, the author degrees were listed incorrectly. The correct author list is as follows:
